# Regulation of mitochondrial morphology and cristae architecture by the TLR4 pathway in human skeletal muscle

**DOI:** 10.3389/fcell.2023.1212779

**Published:** 2023-06-26

**Authors:** Mauricio Castro-Sepulveda, Mauro Tuñón-Suárez, Giovanni Rosales-Soto, Ronald Vargas-Foitzick, Louise Deldicque, Hermann Zbinden-Foncea

**Affiliations:** ^1^ Laboratorio de Fisiología del Ejercicio y Metabolismo, Escuela de Kinesiología, Facultad de Medicina, Universidad Finis Terrae, Santiago, Chile; ^2^ Facultad de Ciencias de la Educación, Universidad San Sebastián, Sede Bellavista, Santiago, Chile; ^3^ Institute of Neuroscience, UCLouvain, Ottignies-Louvain-la- Neuve, Belgium; ^4^ Facultad de Ciencias de la Salud, Universidad Francisco de Vitoria, Madrid, España

**Keywords:** mitochondrial dynamics, skeletal muscle function, mitochondrial nanotunnels, Lipopolysaccharide, TAK^242^, type 2 diabetes

## Abstract

In skeletal muscle (SkM), a reduced mitochondrial elongate phenotype is associated with several metabolic disorders like type 2 diabetes mellitus (T2DM). However, the mechanisms contributing to this reduction in mitochondrial elongate phenotype in SkM have not been fully elucidated. It has recently been shown in a SkM cell line that toll-like receptor 4 (TLR4) contributes to the regulation of mitochondrial morphology. However, this has not been investigated in human SkM. Here we found that in human SkM biopsies, TLR4 protein correlated negatively with Opa1 (pro-mitochondrial fusion protein). Moreover, the incubation of human myotubes with LPS reduced mitochondrial size and elongation and induced abnormal mitochondrial cristae, which was prevented with the co-incubation of LPS with TAK^242^. Finally, T2DM myotubes were found to have reduced mitochondrial elongation and mitochondrial cristae density. Mitochondrial morphology, membrane structure, and insulin-stimulated glucose uptake were restored to healthy levels in T2DM myotubes treated with TAK^242^. In conclusion, mitochondrial morphology and mitochondrial cristae seem to be regulated by the TLR4 pathway in human SkM. Those mitochondrial alterations might potentially contribute to insulin resistance in the SkM of patients with T2DM.

## 1 Introduction

Skeletal muscle (SkM) contributes to a large proportion of whole-body fatty acid uptake and oxidation, as well as to 80% of insulin-stimulated glucose disposal (Baron et al., 1988). Therefore, SkM plays an important role in whole-body metabolic regulation (Pedersen and Febbraio, 2012). In this sense, the mitochondrial function plays an important role in the cell energy supply. In humans, mitochondria occupy >5% of SkM fiber volume (Castro-Sepulveda et al., 2020), and their function has been associated with SkM metabolism and function (Islam et al., 2018).

Mitochondria are dynamic organelles whose morphology (fragmented or elongated) is determined by fusion and fission events (Castro-Sepulveda el al., 2023). SkM mitochondria of subjects with type 2 diabetes mellitus (T2DM) display a fragmented phenotype (Kelley et al., 2002). Moreover, we showed that in human SkM, a mitochondrial connected phenotype was negatively correlated with lipid droplet density and positively correlated with whole-body lipid oxidation (Castro-Sepulveda et al., 2020), which are two variables associated with insulin resistance. In agreement with those *in vivo* results, an *in vitro* study showed that myotubes from insulin resistant patients presented a fragmented mitochondrial phenotype. Treatment of those myotubes with Mdivi-1 drug, a specific inhibitor of mitochondrial fission, restored the mitochondrial connected phenotype, and insulin-stimulated glucose uptake to control levels from healthy volunteers (Kugler et al., 2021). Therefore, mitochondrial morphology plays a role in SkM insulin sensitivity. Elucidating the mechanisms that regulate SkM mitochondrial morphology may reveal important therapeutic targets for metabolic disorders including T2DM.

Toll-like receptor 4 (TLR4), a classical cell pro-inflammatory receptor, is found in the SkM and when activated with LPS induces a local inflammatory response (Reyna et al., 2008; Liang et al., 2013). Increased TLR4 protein expression has been reported in the SkM of subjects with obesity and T2DM, which in turn was associated with insulin resistance (Reyna et al., 2008; Dasu et al., 2010). Therefore, T2DM patients present both fragmented mitochondria phenotype and increased expression of components of the TLR4 pathway in the SkM. However, to date, no study has clearly established any interaction between inflammation and mitochondrial dynamics in the context of insulin resistance. A recent study has shown that incubation of mouse myogenic C2C12 cells with LPS induced fragmented mitochondrial phenotype (Eggelbusch et al., 2022). A similar TLR4-induced fragmented mitochondrial phenotype was found in murine cardiomyocytes (Wu et al., 2018). Together, those results suggest that TLR4 regulates mitochondrial morphology. However, this has not been studied in human SkM. Therefore, we hypothesize that TLR4 regulates mitochondrial morphology in human SkM, which would be involved in the regulation of insulin sensitivity in the context of T2DM.

## 2 Methods

### 2.1 *In vivo* study in healthy human SkM

#### 2.1.1 Subjects

Twelve nondiabetic, nonsmoker men (mean [standard deviation], age, 24.7 [1.5] y; Weight, 75.7 [11.1] kg; body mass index, 24.4 [2.6] kg·m^-2^; glucose, 86.9 [6.8] mg·dl^-1^ insulin, 8.9 [7.6] µUI·ml^-1^; HOMA-IR 2.0 [2.0]; triglycerides, 102 [53] mg·dl^-1^; total cholesterol, 185 [38] mg·dl^-1^; LDL-C, 114 [35] mg·dl^-1^; HDL-C, 51 [14] mg·dl^-1^; VLDL-C, 21 [11] mg·dl^-1^.), without history of cardiovascular, respiratory, or thyroid disease, were included. The sample size was calculated using G-power software, considering the study by Castro-Sepulveda et al. (2020), using the correlation (r = 0.69) between human SKM mitochondrial size (TEM) with whole-body resting RQ, with a power (1—β) of 0.95. The calculated samples size was 10. A 20% of desertion rate or technical problem with the muscle was taken into account for a final sample size of 12 subjects per group. Volunteers were recruited through posters placed at the Finis Terrae University and on massive social media. The volunteers were summoned to a brief interview during which the study protocol was explained to them. In this interview, the inclusion criteria were evaluated and the informed consent was given to and signed by the volunteers. Biopsies from the vastus lateralis of the dominant leg were obtained at the middle point between the anterior superior iliac spine and the patella. The modified Bergström needle technique with suction was used. After local anesthesia (2% lidocaine) was applied, a small incision was made into the skin and fascia, and the biopsy needle was inserted into the muscle. About 100 mg of tissue was withdrawn through manual suction. Excess blood, visible fat, and connective tissue were removed from the tissue. All biopsies were obtained at rest between 8:00 and 9:00 AM. Half of the muscle tissue was immediately frozen in liquid nitrogen and stored at −80°C for Western blotting; the other half was fixed in 2.5% glutaraldehyde for transmission electron microscopy analyses (Castro-Sepulveda et al., 2020). After the biopsies, the doctor gave the health recommendations to the volunteers to treat the incision during the following 56 h. None of the volunteers reported inconveniences. All subjects signed a written informed consent form in accordance with the Helsinki Declaration, and it was approved by the Institutional Review Board at the Universidad Finis Terrae (22-077)-Accredited by SEREMI of Health Exempt Resolution No. 002681/2021.

#### 2.1.2 SkM preparation for transmission electron microscopy (TEM) and image analyses

The SkM biopsies were fixed, and dissected into fiber bundles, washed with 0.1 M sodium cacodylate buffer, stained with 2% osmium tetroxide in 0.1 M sodium cacodylate buffer for 2 h and embedded in Epon resin. Finally, 80-nm sections were examined using a TEM (Tecnai T12 at 80 kV, Philips; Microscopy Facility, Pontificia Universidad Católica de Chile). Mitochondrial morphology was assessed using Fiji/ImageJ software.

#### 2.1.3 Western blotting

SkM samples were homogenized in a lysis buffer as described previously (Castro-Sepulveda et al., 2020). Proteins were separated by SDS-PAGE and transferred to PVDF membranes. The following antibodies were used: Mfn1 (sc-166644-Santa Cruz Biotechnology), Mfn2 (ab56889-Abcam), Opa1 (612606-BD-Biosciences), TLR4 (sc-293072-Santa-Cruz-Biotechnology), Drp1 (sc-101270-Santa-Cruz-Biotechnology), pDrp1 (Ser616, 3455-Cell-Signaling-Technology), Fis1 (ALX-210-1037-Enzo-Life-Sciences), total OXPHOS cocktail (ab110413-Abcam) and GAPDH (2118-Cell-Signaling-Technology). Representative pictures of the blots can be found in Supplementary Figure S1. The Western blotting data for Mfn1-2, Opa1, GAPDH, and TEM images has been previously published (Castro-Sepulveda et al., 2020), but herein we re-analyzed those data differently.

#### 2.1.4 Blood analyses and maximal oxygen uptake

Serum levels of insulin were determined by chemiluminescence and triglycerides, total cholesterol, low-density lipoprotein cholesterol (LDL-C), and high-density lipoprotein cholesterol (HDL-C) by dry chemistry (Bionet, Santiago, Chile). Blood samples were taken ∼7 days prior to the muscle biopsy. Maximal oxygen uptake (VO2max) was determined during an incremental cycle ergometer as previously described (Castro-Sepulveda et al., 2020).

### 2.2 *In vitro* study in human primary myotubes

#### 2.2.1 SkM tissue explants and satellite cell isolation

For this study, the inclusion criteria for the healthy volunteers were: BMI between 18 and 25 kg·m^-2^ and no diagnosis of metabolic syndrome, insulin resistance, or T2DM. For the T2DM group, the inclusion criteria was: having a fasting glycaemia over 126 mg·dl^-1^ or over 200 mg·dl^-1^, 2 h following a 75 g glucose challenge. All subjects signed a written informed consent form in accordance with the Helsinki Declaration. The study was approved by the Institutional Review Board at the Universidad Finis Terrae (22-076)-Accredited by SEREMI of Health Exempt Resolution No. 002681/2021. SkM biopsies of the vastus lateralis were obtained from healthy males (n = 3; age, 31.0 ± 3.6 years; body mass index, 25.2 ± 1.8 kg·m^-2^; HOMA-IR, 1.1 ± 0.3), and T2DM patients (n = 3; age, 42.5 ± 7.5 years; body mass index, 31.5 ± 4.2 kg·m^-2^; HOMA-IR, 3.6 ± 0.2). After the biopsies, the doctor gave the health recommendations to the volunteers to treat the incision with ice gel pack during the following 56 h. None of the volunteers reported inconveniences. A part of the muscle biopsy (∼20 mg) was placed in a 35-mm plate coated with Matrigel and maintained in a growth medium (DMEM, Sigma-Aldrich). Cells were harvested using dispase (BD Biosciences), sub-cultured in growth medium, and then sorted using magnetic activated cell sorting (MACS^©^; Miltenyi Biotec) with magnetic microbeads directly linked to CD56 (purified mouse, anti-human CD56, BD-Biosciences) to isolate satellite cells from other cell populations (Valero-Breton et al., 2020).

#### 2.2.2 Myoblast transfection, differentiation to myotubes, and image analysis

For transfection with mtDsRed plasmid, myoblasts from three healthy and three T2DM volunteers were evenly pooled and incubated at 37°C. The myoblast pool were transfected with mtDsRed plasmid (1 µg) using Lipofectamine 2000 (2 μg, Thermo Fisher Scientific). Healthy myotubes were incubated with DMSO (vehicle, CTRL), LPS (10 µg·ml-1, activator of TLR4) and LPS + TAK^242^ (1 μM, inhibitor of TLR4) for 24 h. Confocal images were captured in live myotubes (Zeiss LSM 880 with Airyscan detection, Microscopy Facility, Pontificia Universidad Católica de Chile). Mitochondrial number and density were quantified using Fiji/ImageJ software.

#### 2.2.3 Myotube preparation for TEM and image analysis

After 24 h of treatment, the myotubes were dissociated with trypsine (Thermo Fisher Scientific), centrifuged, and pellefixed in 2% glutaraldehyde. The fixed pellet was dissected into fiber bundles and analyzed by TEM (Tecnai T12 at 80 kV, Philips; Microscopy Facility, Pontificia Universidad Católica de Chile). Mitochondrial morphology was evaluated as previously described (Vincent et al., 2016; Castro-Sepúlveda et al., 2021a). Example of morphometric analyses in Supplementary Figure S2.

#### 2.2.4 Oxygen consumption

Live myotubes were resuspended in PBS (500 μL) and placed in a gas-tight chamber. Basal oxygen consumption rate (OCR) was measured at 37°C using a Clark electrode (Yellow Springs Instruments) (Castro-Sepúlveda et al., 2021b).

#### 2.2.5 Single-cell fluorescent hexose uptake assay

Myotubes were treated with insulin (100 nM, Actrapid, Novo-Nordisk, Denmark) for 20 min. After, the myotubes were washed and incubated with 2-NBDG (Molecular Probes, Invitrogen, Carlsbad; 300 µM) for 15 min, and transferred to a confocal microscope (Carl Zeiss Pascal 5; Universidad de Chile). Hexose uptake was estimated by comparing intracellular fluorescence with the extracellular signal of live myotubes (Rosales-Soto et al., 2020). The images were quantified by Fiji/ImageJ software.

### 2.3 Statistics

Data are presented as means and standard deviations. Correlations were analyzed using Pearson test. Comparisons between groups were performed using Kruskal–Wallis test and Dunn´s *post hoc* test for multiple comparisons. A two-way ANOVA was used (after logarithmic transformation) to compare 2-NBDG myotube uptake between groups. *p* < 0.05 was considered significant. Prism 7 (GraphPad Software, La Jolla, CA) was used for analyses.

## 3 Results

TLR4 protein expression did not correlate with age, body mass index, or metabolic parameters (Table 1).

**TABLE 1:** Correlation of TLR4 protein expression with age, body mass index, and metabolic parameters.

*n* = 12	r value	*p*-value
Age (y)	−0.27	0.39
Body mass index (kg·m^-2^)	−0.21	0.51
Glucose (mg·dl^-1^)	−0.30	0.34
Insulin (µUl·ml^-1^)	−0.26	0.42
HOMA-IR	−0.25	0.44
VO_2max_ (ml·kg^-1^·min^-1^)	0.09	0.77

HOMA-IR, homeostasis model assessment of insulin resistance; VO2max, maximal oxygen uptake.

**FIGURE 1:**
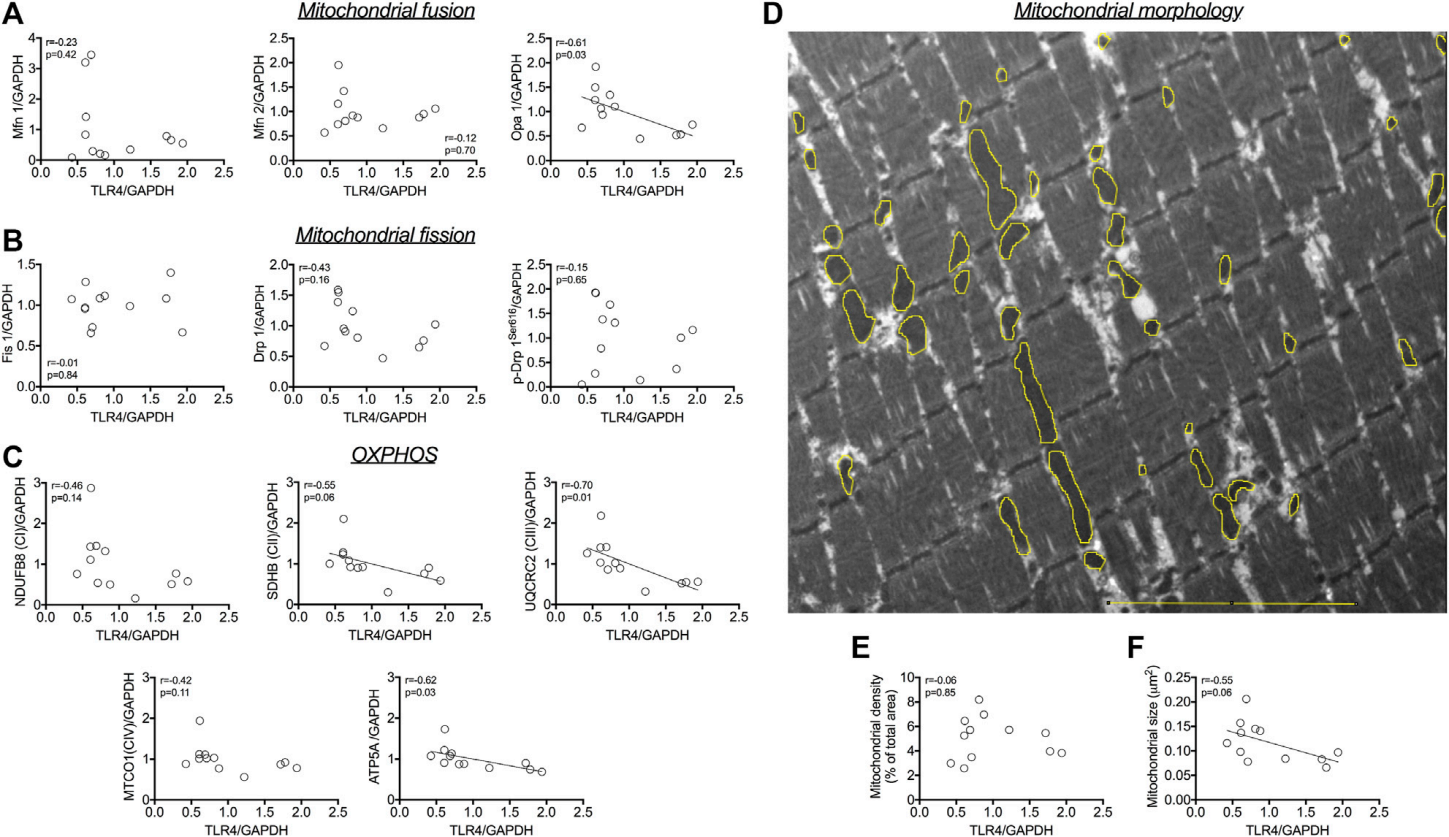
Association between TLR4 protein and **(A)** pro-fusion mitochondrial protein expression (Mfn1, Mfn2 and Opa 1). Association between TLR4 protein and **(B)** pro-fission mitochondrial protein expression (Fis1, Drp1 and Drp1Ser616). Association between TLR4 protein expression and **(C)** OXPHOS proteins **(D)** Representative image of mitochondria in skeletal muscle; in yellow, example of masks drawing over outer mitochondrial membrane to evaluate mitochondrial morphology. Correlation between TLR4 protein expression and **(E)** mitochondrial size, and **(F)** mitochondrial density. Associations were tested using Pearson tests (n = 12). Bar, 5 µm. Associations were tested using Pearson tests (n = 12).

The protein expression of TLR4 did not correlate with outer mitochondrial membrane pro-fusion Mfn1 and Mfn2 (Figure 1A), nor with pro-fission Fis1, Drp1 and Drp1Ser616 (Figure 1B). A negative correlation was detected between the protein expression of TLR4 and the inner mitochondrial membrane pro-fusion Opa1 (r = −0.61; *p* = 0.03; Figure 1A). TLR4 protein expression was negatively correlated with SDHB (complex II; r = −0.55; *p* = 0.06; Figure 1C), UQCRC2 (complex III; r = −0.70; *p* = 0.01; Figure 1C), and ATP5A protein expression (r = −0.62; *p* = 0.03; Figure 1C). Morphometric analysis revealed a negative correlation between TLR4 protein expression and mitochondrial size (r = −0.55; *p* = 0.06; Figures 1D, F), but not with mitochondrial density (r = −0.06; *p* = 0.85; Figures 1E, D).

**FIGURE 2:**
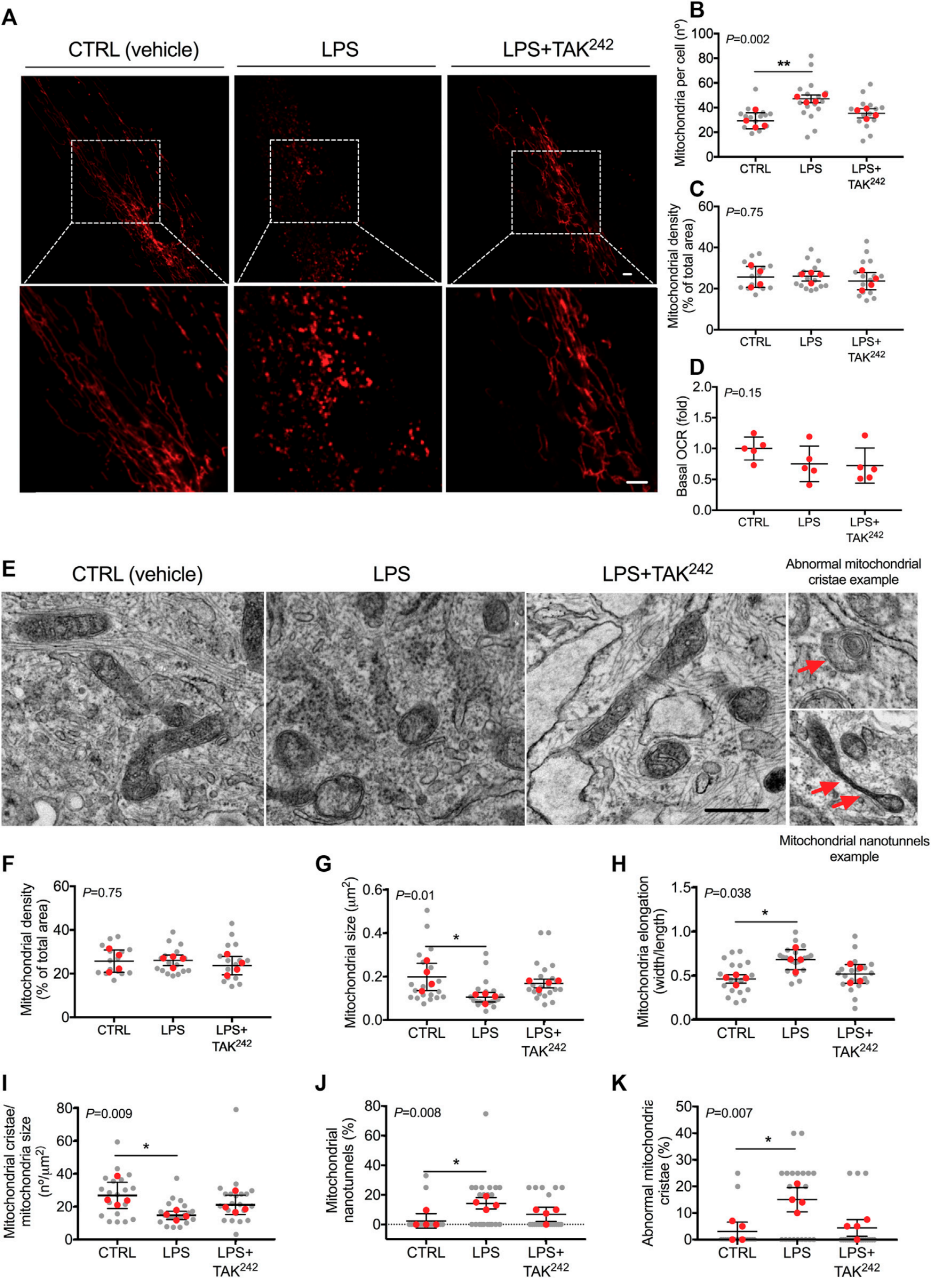
**(A)** Representative image of mitochondria in live human myotubes transfected with mtDsRed (Red) and treated with DMSO (vehicle, CTRL), LPS and LPS + TAK^242^, Bar, 20 µm **(B)** Mitochondrial number per cell **(C)** mitochondrial density and **(D)** basal OCR in live human myotubes treated with vehicle, LPS, and LPS + TAK^242^ **(E)** Representative electron microscopy image of mitochondria in human myotubes treated with DMSO (vehicle, CTRL), LPS, and LPS + TAK^242^, Bar, 1 µm **(E)** Representative image of mitochondria morphology, abnormal mitochondrial cristae and nanotunnels (red arrows) in TEM. Mitochondrial **(F)** density, **(G)** size, **(H)** elongation, **(I)** cristae number, **(J)** nanotunnels and **(K)** abnormal cristae in human myotubes treated with vehicle, LPS and LPS + TAK^242^. n = 4 independent experiments per group; 4-7 myotubes per experiment. The red points in the graphs indicate the mean of the 4 independent experiments. The gray points indicate technical replicates. Data shown in the graphs are means and SD. *p*-value of the Kruskal–Wallis test is given at the top left of each graph. **, *p* < 0.01, *, *p* < 0.05 from the Dunn’s test.

LPS treatment led to a higher mitochondrial number (*p* = 0.008; Figures 2A, B), but did not modify mitochondrial density (Figures 2A, C) nor myotube basal respiration (Figure 2D), compared to CTRL. When myotubes were co-treated with LPS and TAK^242^, no differences were found in mitochondrial number (Figure 2B) compared to CTRL. We confirmed by TEM that LPS altered the mitochondrial morphology (Figures 2E–H). As mitochondrial fusion/fission imbalances may alter mitochondrial cristae structure (Otera et al., 2016; Castro-Sepúlveda et al., 2021a), we evaluated the effects of LPS and TAK^242^ treatment on cristae structure in human myotubes (Figures 2E, I–K). LPS reduced the mitochondrial cristae number (*p* = 0.012; Figure 2I) and increased the percentage of mitochondrial nanotunnels (*p* = 0.011; Figure 2J) and abnormal mitochondrial cristae (*p* = 0.019; Figure 2K), which were all mitigated by the addition of TAK^242^ to LPS.

**FIGURE 3:**
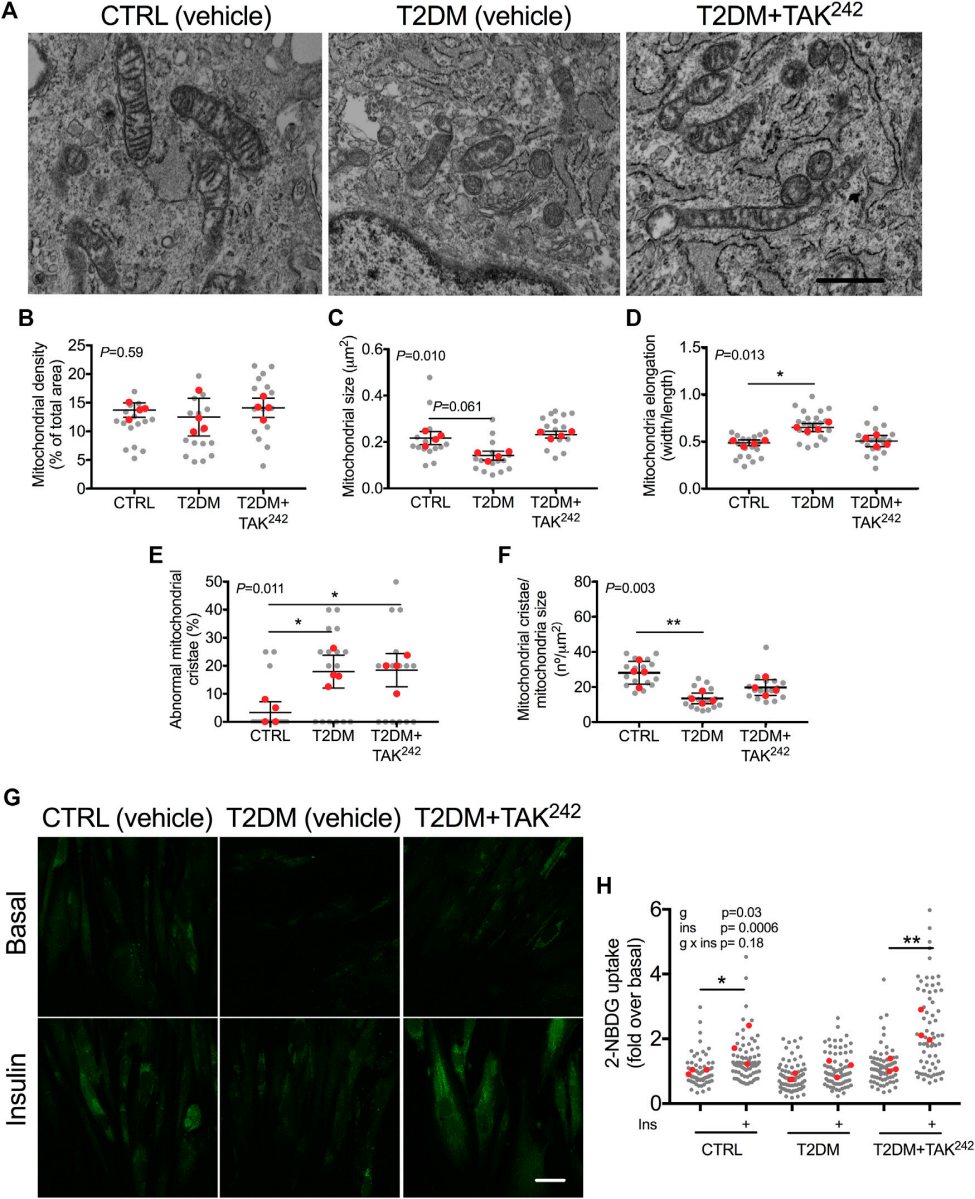
**(A)** Representative image of mitochondria in human myotubes treated with DMSO (vehicle, CTRL), myotubes from people with type 2 diabetes mellitus (T2DM) and T2DM myotubes treated with TAK^242^. Mitochondrial **(B)** density **(C)** size **(D)** elongation **(E)** abnormal cristae and **(F)** cristae number in CTRL, T2DM and T2DM + TAK^242^ myotubes. *p*-value of the Kruskal–Wallis test is given at the top left of each graph. *, *p* < 0.05 and **, *p* < 0.01 from the Dunn’s test. **(G)** Representative image of 2-NBDG (green) and **(H)** 2-NBDG uptake in CTRL, T2DM and T2DM + TAK^242^ myotubes in basal and insulin-stimulated conditions. *p*-values of the 2-way ANOVA are given at the top left. *, *p* < 0.05 and **, *p* < 0.01 from the *post hoc* Dunn’s test. n = 3-4 independent experiments per group; 4–10 myotubes per experiment. The red points in the graphs indicate the mean of the 4 independent experiments. The gray points indicate technical replicates. Data shown in the graphs are means and SD. Bar in A, 1 μm; Bar in F, 10 µm.

The role of TLR4 in mitochondrial disturbances was then investigated in a clinical model of inflammation such as myotubes from T2DM patients. T2DM myotubes had less elongated mitochondria (*p* = 0.028; Figures 3A–D), a higher percentage of abnormal mitochondria cristae (*p* = 0.047; Figures 3A, E), and a lower number of mitochondrial cristae (*p* = 0.008; Figures 3A, F) than health CTRL myotubes, which were reversed when T2DM myotubes were treated with TAK^242^. As it has previously been evidenced that restoration of the connected mitochondrial phenotype in myotubes from insulin-resistant patients by a mitochondrial anti-fission drug restored insulin-stimulated glucose uptake (Kugler et al., 2021), we evaluated whether restoration of the connected mitochondrial phenotype by TAK^242^ contributed to insulin-stimulated glucose uptake in myotubes of patients with T2DM. As expected, we found that insulin increased 2-NBDG uptake in CTRL (*p* = 0.022) but not in T2DM myotubes (Figures 3G, H). TAK^242^ treatment restored insulin-stimulated 2-NBDG uptake in T2DM myotubes (*p* = 0.006; Figures 3G, H). We found no differences between the CTRL, T2DM, nor T2DM + TAK^242^ groups in basal conditions (-Ins). In insulin-stimulated conditions (+Ins), we found differences between CTRL and T2DM (*p* = 0.052), T2DM and TAK^242^ (*p* = 0.02), but not between CTRL and TAK^242^ (*p* = 0.26).

## 4 Discussion

We hypothesized in this study that in human SkM, mitochondrial morphology could be regulated by the TLR4 pathway, which could in turn contribute to changes in insulin resistance. The main findings were that: 1) in human SkM *in vivo*, TLR4 protein expression was negatively correlated to Opa1 protein expression and mitochondrial size; 2) in human myotubes, LPS-induced activation of TLR4 increased mitochondrial fragmented phenotype and increased the percentage of abnormal mitochondrial cristae and nanotunnels, which was reversed when TAK^242^ was added to LPS; and 3) T2DM myotubes displayed a mitochondrial fragmented phenotype, and abnormal mitochondrial cristae structure compared to healthy myotubes, which was partially rescued when T2DM myotubes were treated with TAK^242^; 4) TAK^242^ restored the lower insulin-stimulated glucose uptake in T2DM compared to healthy myotubes; potentially due to restoration of the connected mitochondrial phenotype as previously evidenced (Jheng et al., 2012; Kugler et al., 2021).

Previous studies have shown that the TLR4 pathway may regulate mitochondrial morphology in *vitro* muscle models and in murine cardiac muscle. C2C12 cells treated with LPS for 24 h increased the mitochondrial fragmented phenotype (Eggelbusch et al., 2022). In mice, intraperitoneal injection of LPS twice a week for 5 weeks induced a mitochondrial fragmented phenotype in the cardiac muscle and lower Opa1 and Mfn1 protein expression (Wu et al., 2018). In line with this, we found a positive correlation between TLR4 protein expression in skeletal muscle and the mitochondrial fragmented phenotype. Interestingly, we found that higher TLR4 protein expression was associated with lower Opa1 protein expression in human SkM, as was found in murine cardiomyocytes treated with LPS (Wu et al., 2018). Opa1 is a pro-fusion mitochondrial protein and plays a role in mitochondrial cristae morphology (Cartes-Saavedra et al., 2023). Therefore, Opa1 might mediate TLR4-induced inflammation to regulate mitochondrial fusion and mitochondrial cristae. Finally, TLR4 protein expression was negatively correlated with OXPHOS (CII, CIII and CV) protein expression. This suggests that TLR4 could regulate the oxidative capacity in human SkM through the regulation of mitochondrial morphology. Regarding the mechanism by which the activation of TLR4 by LPS regulates Opa1 and OXPHOS, it has recently been shown in renal tubular epithelial cells that LPS decreased deacetylation of i-AAA protease (YME1L1), an upstream regulatory molecule of OPA1 (Jian et al., 2023). Therefore, we speculate that chronic low-grade inflammation in young subjects could decrease the OPA1 protein expression through YMEIL1. The chronic low-grade inflammation-induced decrease in Opa1 may be an initial step in the development of metabolic alterations in the SkM of young subjects, but this requires further investigation. Another potential mediator of LPS-induced changes in mitochondrial structure alterations in SkM is sarcopilin, however, more studies are needed to confirm this (Bal et al., 2021).

In SkM, mitochondrial cristae density is associated with metabolic capacity in humans (Castro-Sepulveda et al., 2023) and thermogenesis in mice (Bal et al., 2017). However, it is not fully understood how mitochondrial cristae density or architecture is regulated. A potential regulator is inflammation, as we showed that COVID-19 patients with severe infection, characterized by higher systemic inflammation, had a lower mononuclear cell mitochondrial cristae density (Castro-Sepulveda et al., 2022). Our study in human myotubes is the first to evaluate the effects of LPS on mitochondrial cristae architecture. Our results showed that LPS treatment induced abnormal mitochondrial cristae, mitochondrial nanotunnels, and reduced mitochondrial cristae density, which was reversed upon LPS co-treatment with TAK^242^. Mitochondrial nanotunnels were previously described in human SkM myopathy (Vincent et al., 2016), and inflammation is one of the characteristics of myopathies. Emerging evidence suggests that mitochondrial nanotunnels are generated by immobilized mitochondria (Vincent et al., 2017). One could hypothesize that TLR4-induced inflammation could immobilize mitochondria, but this must be investigated and confirmed in future studies. Calcium release from the endoplasmic reticulum and shuttle to mitochondria could contribute to the regulation of mitochondrial cristae by TLR4 (Swalsingh et al., 2022). In summary, our results show that LPS induced a mitochondrial fragmented phenotype and induced alterations in mitochondrial cristae, which was reversed upon LPS co-treatment with TAK^242^ in human myotubes.

Cultured human myotubes from T2DM patients maintain the same phenotype characteristics as the donor with diabetes, such as the inflammatory state (Gaster et al., 2002; Hansen et al., 2004). Hence, we evaluated the effects of TAK^242^ on mitochondrial morphology and cristae, and insulin resistance in myotubes from T2DM patients. Our study is the first to evaluate mitochondrial morphology and mitochondrial cristae in human T2DM myotubes. T2DM human myotubes had a fragmented mitochondrial phenotype, and abnormal mitochondrial cristae structure, which was partially rescued upon TAK^242^ treatment. Our study in T2DM myotubes confirmed the role of TLR4 and inflammation in the regulation of mitochondrial morphology and mitochondrial cristae architecture. Indeed, the treatment with TAK^242^, a specific inhibitor of TLR4 with anti-inflammatory effect, restored the mitochondrial morphology and partially the architecture of the mitochondrial cristae in T2DM myotubes. Interestingly, the reduced insulin-stimulated glucose uptake in T2DM vs. control myotubes was restored as well after TAK^242^ treatment, together with the restoration of the connected mitochondrial phenotype. A previous study in human myotubes from healthy subjects showed that LPS treatment for 12 h reduced insulin-stimulated glucose uptake (Liang et al., 2013). With respect to the role of mitochondrial morphology in insulin resistance, it has previously been found that both genetic and pharmacological inhibition of mitochondrial fission restored insulin-stimulated glucose uptake in several models of insulin resistance such as lipotoxicity in C2C12, high-fat diet in mice and myotubes from humans with insulin resistance (Jheng et al., 2012; Kugler et al., 2021). Finally, the activation of TLR4 might reduce insulin-stimulated glucose uptake by dysregulation of mitochondrial morphology in primary cardiomyocytes from rats (Parra et al., 2014), which our results confirm in human myotubes. Together those results strongly suggest that activation of the TLR4 pathway induces a mitochondrial fragmented phenotype, which decreases insulin sensitivity.

We based the choice of the concentration and duration for LPS and TAK^242^ on a previous study in a cardiomyocyte cell line using a concentration of 10 µg LPS·ml^-1^ and a duration of incubation of 24 h, conditions that were able to induce a fragmented mitochondrial phenotype (Wu et al., 2018). In a recent study in C2C12 myotubes, lower LPS concentrations together with a shorter incubation duration (100 ng LPS·ml^-1^ for 2 h) induced a fragmented mitochondrial phenotype as well (Eggelbusch et al., 2022). Finally, in human myotubes, incubation of 50 pg LPS·ml^-1^ and 500 ng·ml^-1^ for 2, 6, 12, and 24 h all induced a similar decrease in fatty acid oxidation (Frisard et al., 2010). Altogether, those results suggest that a wide range of LPS concentrations have similar effects on muscle metabolism *in vitro*. For TAK^242^ concentrations, it seems that a consensus has been reached in the literature for using 1 µM as it is consistently efficient at reducing the harmful effects of LPS or other toxins *in vitro* and *in vivo* (Takashima et al., 2009; Ono and Sakamoto, 2017; Ono et al., 2020).

In conclusion, mitochondrial morphology and mitochondrial cristae seem to be regulated by the TLR4 pathway in human SkM. Those mitochondrial alterations might potentially contribute to insulin resistance in the SkM of patients with T2DM. Both the TLR4 pathway and downstream mitochondrial alterations could therefore be of potential interest in the development of pharmacological treatments against insulin resistance.

## Data Availability

The original contributions presented in the study are included in the article/Supplementary Material, further inquiries can be directed to the corresponding authors.
