# Supplementary material for: Regulation of mitochondrial morphology and cristae architecture by the TLR4 pathway in human skeletal muscle
Source: Front Cell Dev Biol. 2023 Jun 26;11:1212779. doi: 10.3389/fcell.2023.1212779 (PMC10332154; doi:10.3389/fcell.2023.1212779)
Supplement: Supplementary file 1 [file Table1.DOCX]

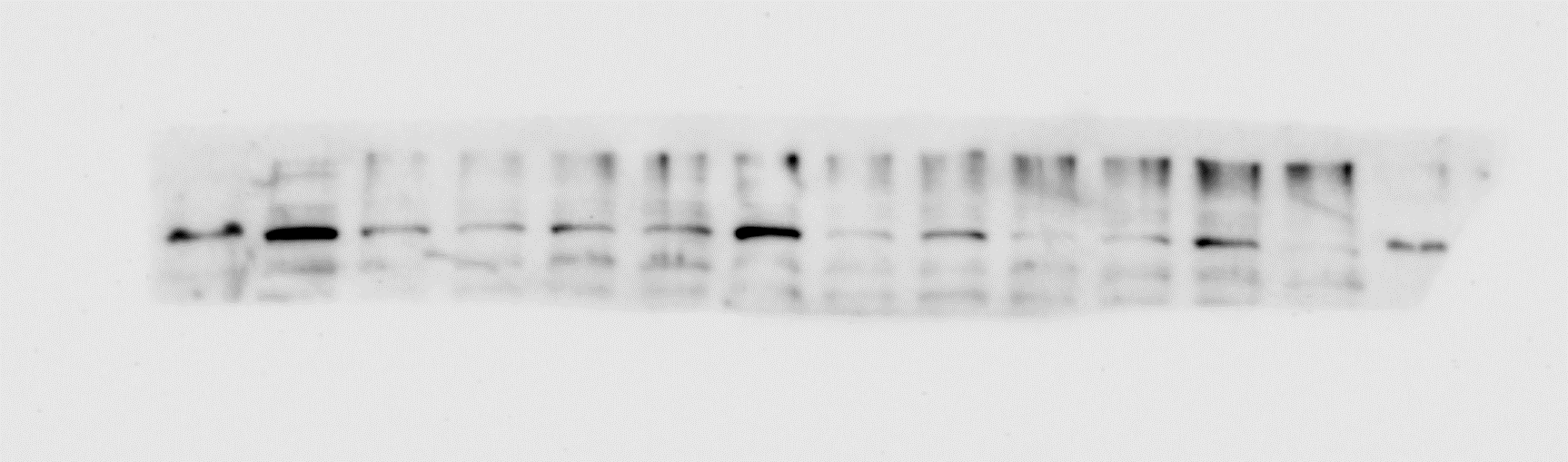

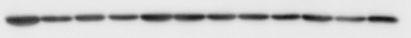

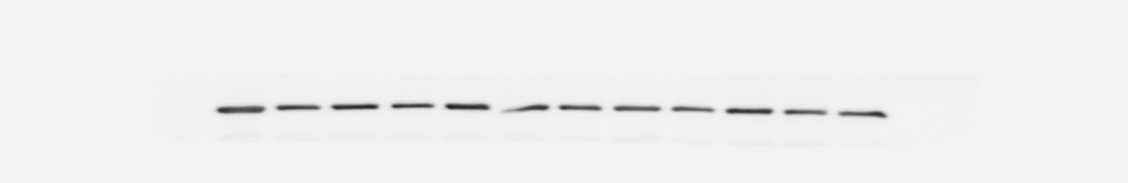

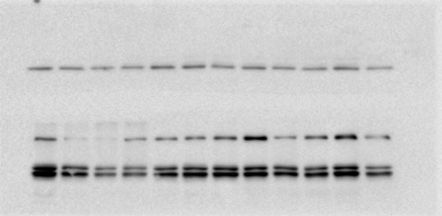

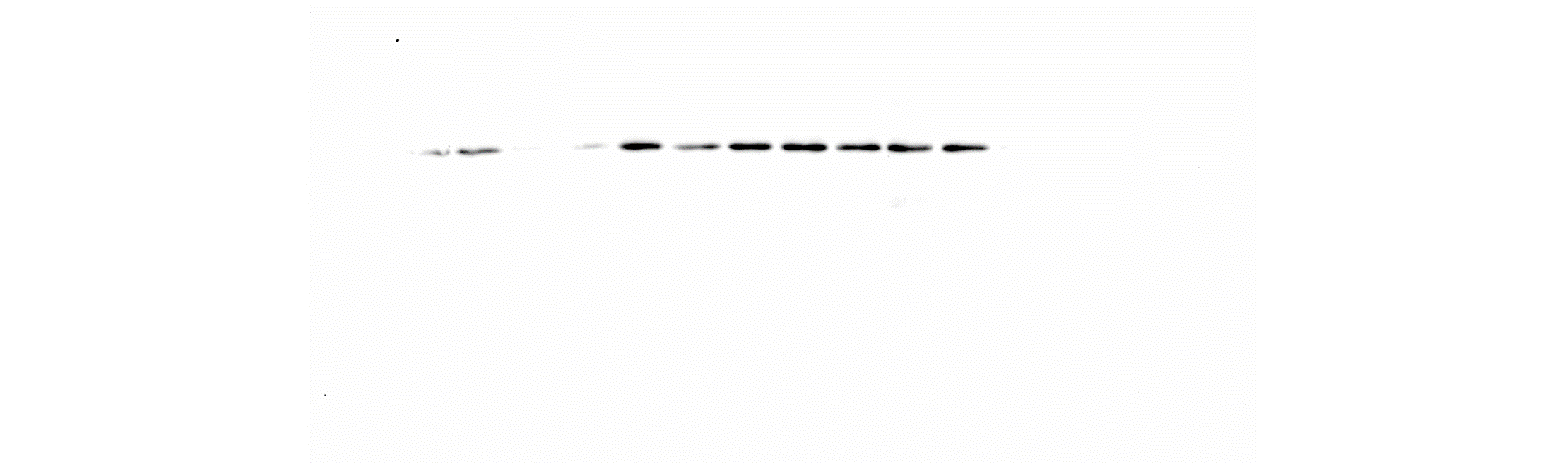

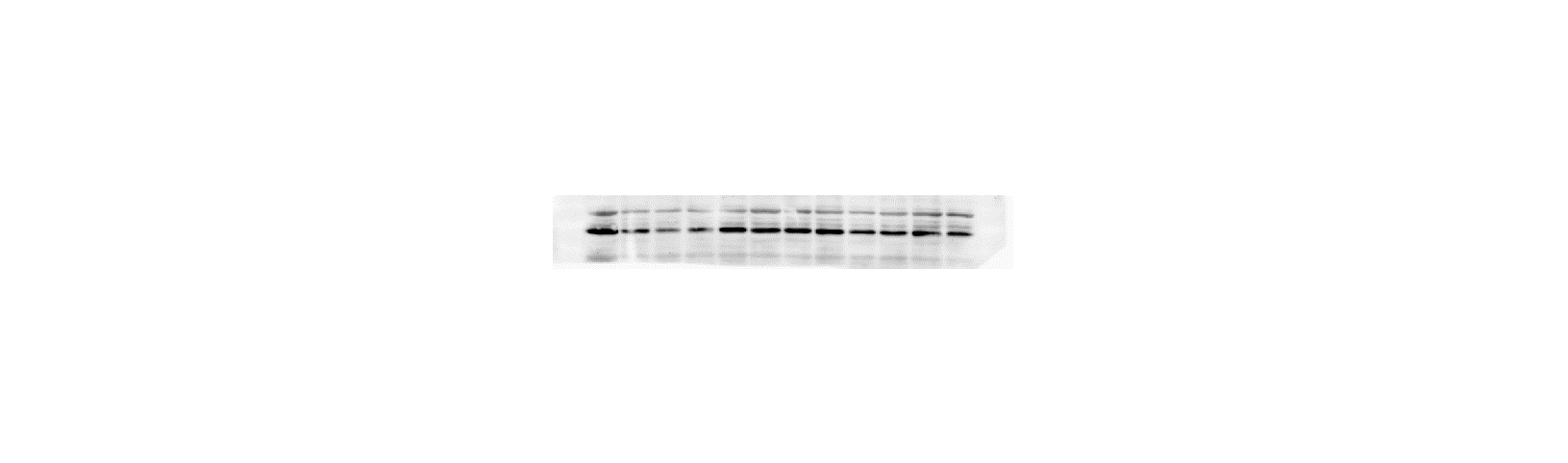

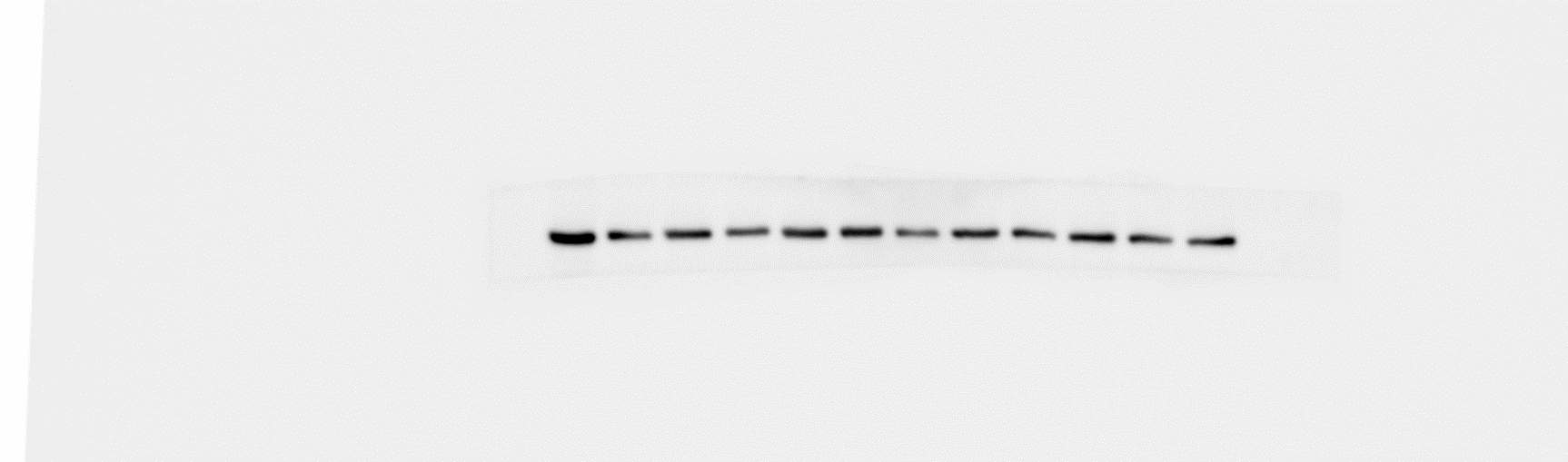


~75kDa

Mfn 1

~35kDa

GAPDH111

~75kDa

Mfn 2

CIV

GAPDH

~35kDa


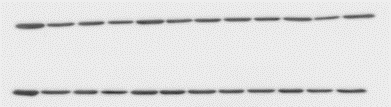


GAPDH


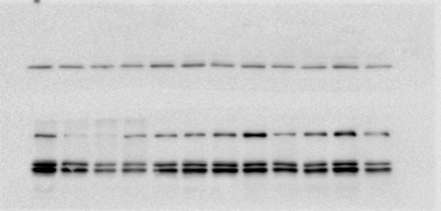


~80-100kDa

Opa 1


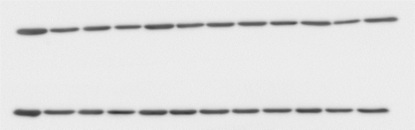


~35kDa

GAPDH

~80kDa

p-Drp1^Ser616^

~80kDa

t-Drp1

~35kDa

GAPDH


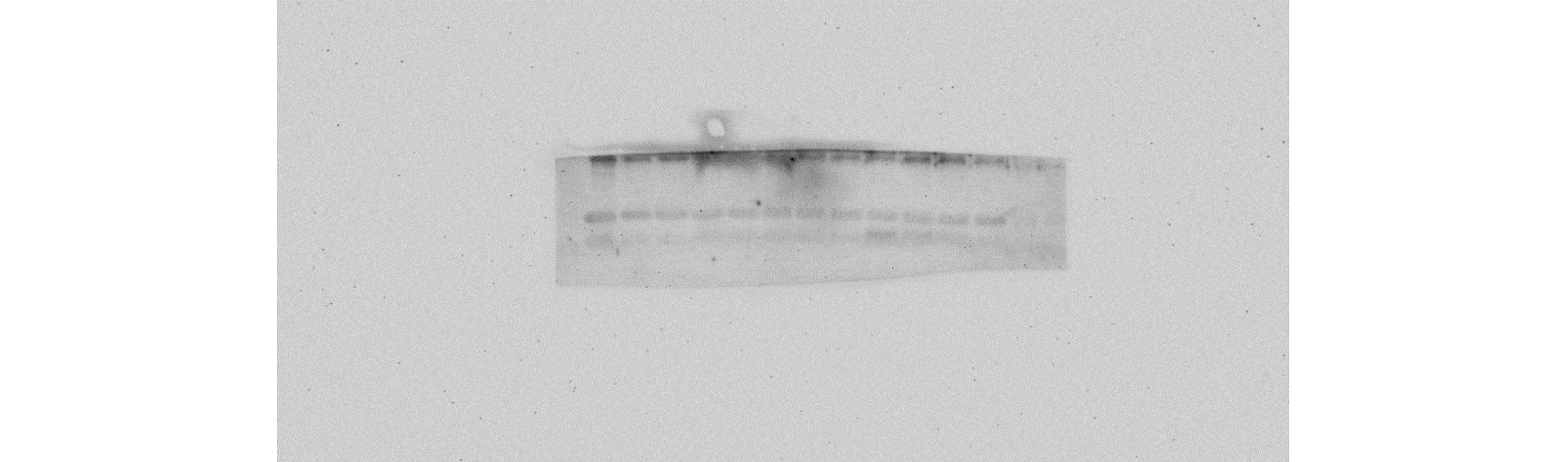


~15kDa

Fis 1

~35kDa

GAPDH


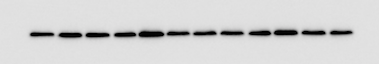


~110kDa

TLR4


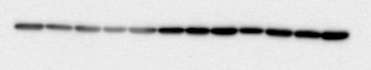


~35kDa

GAPDH


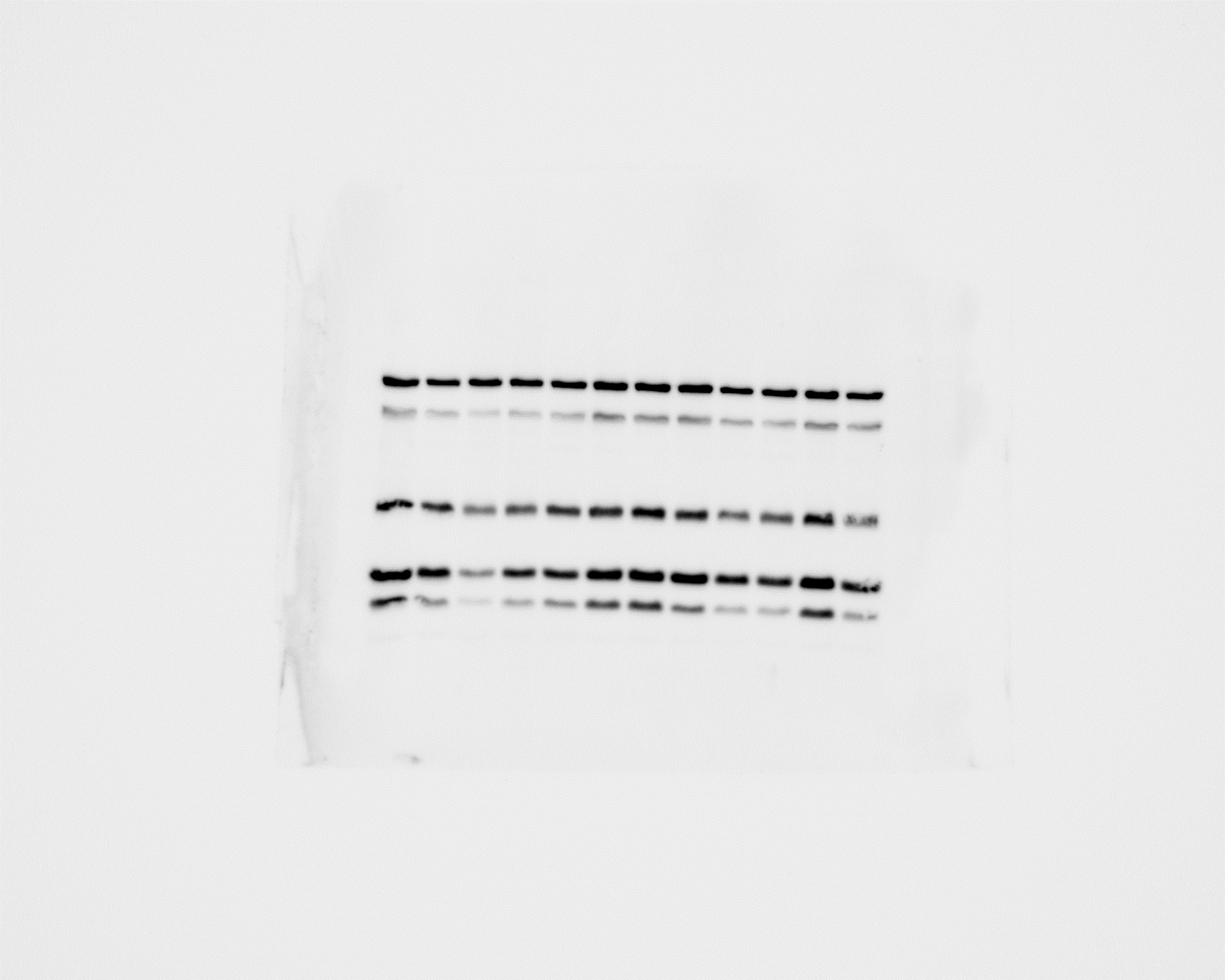


~55kDa

~45kDa

CV

CIII

~40kDa

~30kDa

CII

~20kDa

CI


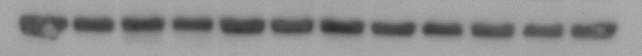


~35kDa

**Supplementary Figure 1** Representative pictures of the blot from healthy volunteers (figure 1).
