# Supplementary material for: Regulation of mitochondrial morphology and cristae architecture by the TLR4 pathway in human skeletal muscle
Source: Front Cell Dev Biol. 2023 Jun 26;11:1212779. doi: 10.3389/fcell.2023.1212779 (PMC10332154; doi:10.3389/fcell.2023.1212779)
Supplement: Supplementary file 2 [file Table2.DOCX]

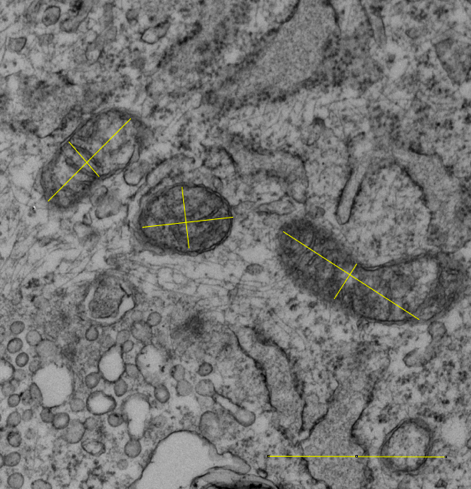

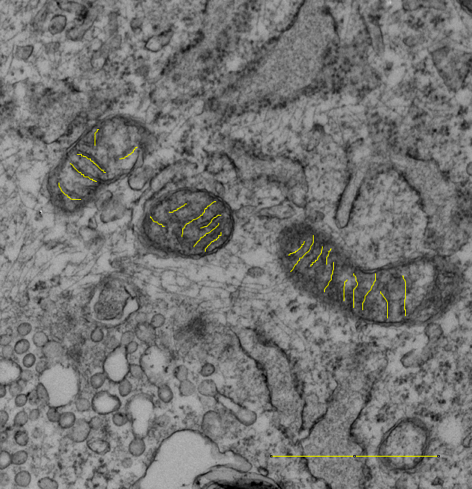
A B C


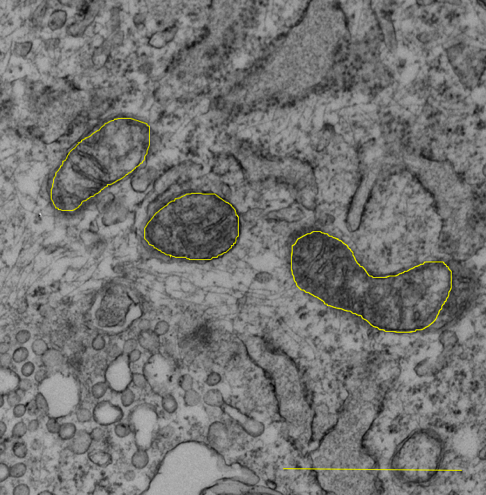


**Supplementary Figure 2** Morphometric analyses of (A) mitochondrial size, (B) mitochondrial cristae number, and (C) mitochondria elongation (width/length) from TEM images of human myotube. Bar, 1 µm.
